# Bench Testing of a New, Semirigid, Saddle-Shaped, Complete Mitral Annuloplasty Ring Designed to Circularize During Transcatheter Mitral Valve-in-Ring Procedures

**DOI:** 10.1016/j.shj.2025.100791

**Published:** 2025-12-30

**Authors:** Keith B. Allen, Daniel J. Romary, Elizabeth A. Grier, Chetan P. Huded, Duc T. Pham, Douglas R. Johnston, Panos N. Vardas, John R. Davis, Adnan K. Chhatriwalla

**Affiliations:** a Division of Cardiac Surgery, St. Luke’s Mid America Heart Institute, Kansas City, Missouri, USA; b Division of Cardiac Surgery, Northwestern University Feinberg School of Medicine and Bluhm Cardiovascular Institute at Northwestern Medicine, Chicago, Illinois, USA; c Division of Cardiology, St. Luke’s Mid America Heart Institute, Kansas City, Missouri, USA; d Division of Cardiac Surgery, University of Alabama at Birmingham, Birmingham, Alabama, USA

**Keywords:** Annuloplasty ring, Lifetime management of valvular heart disease, Mitral valve repair, Transcatheter mitral valve replacement, Valve-in-ring

## Abstract

• A new mitral annuloplasty ring was purpose-built for transcatheter valve-in-ring.
• Bench testing confirmed anchoring of transcatheter valves inside the ring.
• Balloon filling volumes are provided for several sizes of valve-ring pairs.
• Surgeons should consider future intervention when selecting an annuloplasty device.
• Heart team collaboration can optimize lifetime management of mitral valve disease.

Although mitral valve repair (MVr) is the preferred treatment for mitral regurgitation, MVr durability is dependent upon the underlying etiology of mitral regurgitation and surgical technique.^1^ Many patients with failed surgical MVr are now older with mounting comorbidities, including increased frailty, that make them less-than-ideal candidates for traditional reoperation. In the current era, these patients are frequently evaluated by a multidisciplinary heart team to determine their best treatment option. Transcatheter mitral valve-in-ring (TMViR) is often considered using existing transcatheter aortic valves in the mitral position. Unfortunately, certain characteristics of the annuloplasty ring, including deformability, completeness of the ring, and three-dimensional (3D) geometry, can affect the suitability for TMViR.^2^

A recently Food and Drug Administration–approved annuloplasty ring, the Genesee TransForm McCarthy Mitral Annuloplasty Ring (Genesee BioMedical, Inc., Denver, CO) was purpose-built to optimize acute MVr results and facilitate potential future valve-in-ring (ViR). Semirigid and saddle-shaped at baseline, the TransForm ring conforms to the natural shape of the mitral annulus with a 4:3 commissure-to-commissure–to–anterior–posterior ratio. It accomplishes this with anterior and posterior stiffeners to achieve the 3D shape and size, and a silicone core at the commissures that allows the ring to stretch to accommodate a transcatheter heart valve (THV). It is a complete ring rather than a band, so it provides a better landing zone than an incomplete ring. Barium-impregnated silicone allows visibility to aid in TMViR procedures. The design at the commissures uniquely allows for transformation from the “D” shape (4:3 ratio) to an “O” shape (1:1 ratio) during TMViR.

We sought to provide the first report on the suitability of the TransForm ring for TMViR and determine the optimal combination of THV sizes with various sizes of the TransForm ring in a bench-top study.

Specifically, bench testing was performed on size 24-32 mm TransForm rings (n = 1 per ring size) using 26- or 29-mm balloon expandable THVs (SAPIEN 3 Transcatheter Aortic Valve; Edwards Lifesciences, Irvine, CA) to simulate TMViR. To replicate in vivo valve deployment, the balloon was hand-inflated to its nominal pressure inside the TransForm ring without the use of a high-pressure inflation. Optimal anchoring and circularization were determined visually and subjectively by a cardiac surgeon (K.B.A.) and interventional cardiologist (E.A.G.), specifically noting the degree of conformity of the ring around the valve system. An acceptable fit was defined as complete expansion of the valve with a circularized ring. There could be no slippage of the ring around the valve and no gaps between the ring and the valve where paravalvular leak could occur. If deemed inadequate, 2 to 6 mL of additional volume was added to the transcatheter delivery balloon to determine the appropriate volume for optimal conformity.

The ring transformed from the resting “D” appearance and 3D saddle shape of the native mitral annulus to a circular “O” shape corresponding to the THV outer diameter in all tests. Figure 1 summarizes the results of the bench testing and provides an easy reference for determining the optimal TMViR configuration. For the 24-mm TransForm ring, a 26-mm THV fit well at nominal volume, whereas a 26-mm ring required a 26-mm valve with an additional 2 mL of volume to achieve a good fit. The 28-mm ring matched with a 29-mm valve at nominal volume, whereas a 30-mm ring required a 29-mm valve with 3 mL of additional volume added to the delivery balloon. When testing a 32-mm ring, a 29-mm THV was insufficiently anchored despite adding an additional 6 mL of volume to the delivery balloon. Although we do not add more than 6 mL of additional volume to a 29-mm SAPIEN 3 valve in clinical practice, in this bench test, we added 7 mL of additional volume, which resulted in balloon rupture.

**Figure 1:**
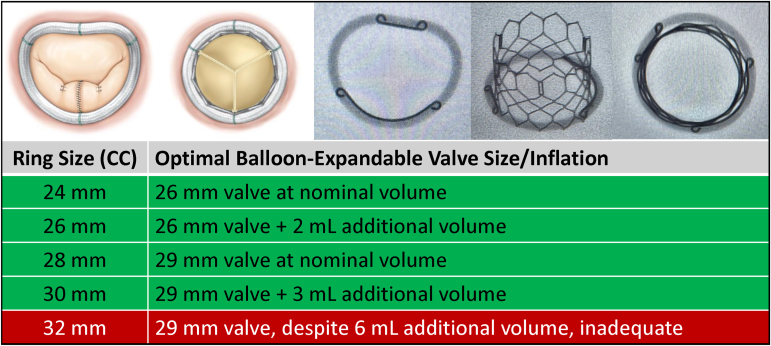
The Genesee TransForm McCarthy Mitral Annuloplasty Ring is a semirigid, saddle-shaped ring with anterior and posterior stiffeners and a silicone core that allow it to transform from “D” to “O” shaped when accepting a transcatheter valve. Corresponding TransForm ring and SAPIEN 3 transcatheter valve pairs are listed with optimal inflation volume from bench testing.

Our findings demonstrate that the TransForm ring does indeed circularize and can anchor a balloon-expandable THV to facilitate TMViR. Second, the results provide a guide for the appropriate valve-ring match and filling volume. We also demonstrated that, for currently approved aortic THVs in the United States, only TransForm rings up to size 30 mm were suitable on a bench-top test given that the largest valves are 29 mm. Theoretically, larger dedicated THVs (i.e. Tendyne [Abbott, Abbott Park, IL]) would be a better fit for large rings.

Lifetime management of patients with valve disease has become increasingly important with an evolution toward the use of more bioprosthetic surgical and transcatheter valves, particularly in younger patients, where a second valve procedure is more likely to be necessary.^3^ Selecting an implant that can accommodate a less invasive transcatheter solution if that index procedure fails is invaluable.

Aortic valve replacement surgeons often alter their technique or choose valves that can facilitate and optimize future valve-in-valve transcatheter aortic valve replacement.^4^^,^^5^ Mitral valve surgeons must also consider the lifetime management of patients undergoing MVr, since an unsuitable choice of annuloplasty ring can disqualify a patient from future TMViR. A mitral ring that meets the surgeon’s standard for an excellent MVr and is designed to facilitate a future TMViR procedure (should that repair fail) is an important tool in a surgeon’s armamentarium.

This study was conducted in a bench-top format with a small number of devices undergoing qualitative assessment. However, it serves as proof of concept for future studies in physiologic environments using larger valves or dedicated transcatheter mitral valve replacements in development. We hope it also encourages continued discussion among the heart team regarding lifetime management and the importance of the index procedure. Finally, the TransForm concept could also be applied to future innovations in tricuspid annuloplasty, as ViR in existing tricuspid rings is even more limited.

Overall, bench testing on a new, saddle-shaped, semirigid complete mitral annuloplasty ring with necessary attributes for durable surgical MVr also demonstrated that the purpose-built ring can potentially facilitate future TMViR. Interventional cardiologists and surgeons should ensure appropriate volume is instilled in balloon-expandable TMViR interventions to effectively seat the valve inside the ring. Cardiac surgeons should be mindful of possible future transcatheter interventions when selecting their mitral annuloplasty device.

## Funding

The authors have no funding to report.

## Ethics Statement

Responsible Conduct of Research principles were followed during this study. Given the bench-testing nature of the project, it was exempt from IRB/IACUC oversight.

## Disclosure Statement

Keith B. Allen reports institutional research grants, proctoring, and consulting with Edwards Lifesciences, Medtronic, and Abbott with all payments to the institution and none to him personally. Elizabeth A. Grier is a consultant for Medtronic. Chetan P. Huded reports institutional research grants, proctoring, and consulting with Medtronic and Boston Scientific. Duc T. Pham is a consultant for Abbott, Medtronic, and AbioMed. Douglas R. Johnston is a consultant for Abbott, Artivion, Edwards Lifesciences, LivaNova, and Terumo. Panos N. Vardas is a consultant for Medtronic. Adnan K. Chhatriwalla reports institutional research grants, proctoring, and consulting with Edwards Lifesciences, Medtronic, Abbott, and Boston Scientific.

The other authors had no conflicts to declare.
